# An irregular atrial tachycardia: What is the underlying mechanism?

**DOI:** 10.1007/s12471-017-1051-7

**Published:** 2017-10-27

**Authors:** S. Pagano, G. Aguglia, D. Noto, M. Averna

**Affiliations:** 1 Sezione di Medicina Interna e Malattie Metaboliche, Dipartimento di Medicina Interna e Specialistica, DIBIMIS, Università di Palermo, Palermo, Italy; 2 Dirigente Medico presso MCAU, ARNAS Civico, Palermo, Italy

A 66-year-old male patient with a history of rheumatoid arthritis, coronary artery disease, hypothyroidism, end-stage renal disease and receiving haemodialysis presented to the emergency department with palpitations. His medication was acetylsalicylic acid, pantoprazole, levothyroxine, and paracetamol and ibuprofen as needed. Laboratory test results showed a mild anaemia and serum potassium levels within the upper limit of normal. A 12-lead electrocardiogram was recorded (Fig. 1). The electrocardiogram shows narrow QRS complexes (0.08 sec) with a QS morphology in leads V1 through V4 consistent with an old antero-septal wall myocardial infarction. The rhythm is irregular at an average rate of 79 beats per minute with groups of beatings separated by pauses. However, the underlying rhythm is not a sinus rhythm.

**Fig. 1:**
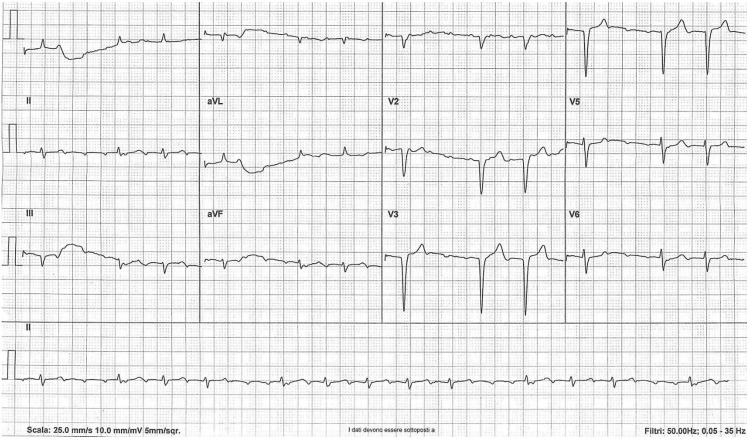
The irregular atrial tachycardia

## Question

What is the mechanism?

## Answer

You will find the answer elsewhere in this issue.

